# Reparatory

**Published:** 1867-09

**Authors:** 


### REPARATORY.

Our friend Dr. Arrington thinks we did him injustice in our "one more unfortunate" article in the August number. We are sorry. He states that he did not urge, as a claim to favor, that Lamm's gold could be used under saliva. We heard that claim repeatedly urged in its favor at the Boston meeting; and were honestly believing that he pressed this claim. But it seems we were mistaken; and we are glad of it. As he is opposed to having the gold thus *baptized*, we had no right to call him its "godfather," and hereby retract the misnomer. Whenever it is claimed that wet fillings are good, the inexperienced are led astray, the lazy and unprincipled take courage, patients are deceived, and teeth are lost. To prevent this was our sole aim in that "unfortunate" editorial. W.

